# Insect Fauna of Human Cadavers in Tehran District

**Published:** 2017-09-08

**Authors:** Fahimeh Talebzadeh, Masoud Ghadipasha, Jaber Gharedaghi, Naser Yeksan, Kamran Akbarzadeh, Mohammad Ali Oshaghi

**Affiliations:** 1 Department of Medical Entomology and Vector Control, School of Public Health, Tehran University of Medical Sciences, Tehran, Iran; 2 Iranian Legal Medicine Organization, Tehran, Iran

**Keywords:** Forensic entomology, Blow fly, Post-mortem interval, *Chrysomya albiceps*, Iran

## Abstract

**Background:**

Entomological data can provide valuable information for crime scene investigations especially in post-mortem interval (PMI) estimation. This study performed to determine insect fauna of human corpses in Tehran district.

**Methods:**

Insect specimens were collected from 12 human cadavers during spring and summer 2014 and were identified using morphological characteristics.

**Results:**

Four fly species including two blowflies *Chrysomya albiceps* and *Lucilia sericata* (Calliphoridae), one flesh fly *Sarcophaga argyrostoma* (Sarcophagidae), and one phorid fly *Megaselia scalaris* (Phoridae) and a beetle *Dermestes maculatus* (Dermestidae) was observed on the human cadavers. *Chrysomya albiceps* was the most dominant species on the corpses temporally and spatially.

**Conclusion:**

*Chrysomya albiceps* was the most dominant insect species on human cadavers in the area study spatio-temporally. The data make *C. albiceps* as a valuable entomological indicator for PMI estimation in Tehran and other parts of the country. However, further biological and ecological data such as its behavior, life tables, and consistent developmental time should be investigated when establishing a PMI in the region.

## Introduction

Various insects and arthropods attract to the specific decomposition stage of human or animal carcass while majority of them colonize for only a limited time (1). Forensic entomology families of the order Diptera have the greatest importance because of their exclusive behavior in early arriving and making abundant larvae on human cadavers (2, 3).

Members of the family Calliphoridae are the first group of insects that attack a corpse within minutes after death (3, 4). Additionally, members of the family Sarcophagidae often colonize in corpse around the world especially in tropical and subtropical countries (3). Thus, they are also very useful in crime scene investigations especially in post mortem interval (PMI) estimation (3). PMI calculations are used for various goals including criminal items, trace of transport of the corpse after death, correlation defiant with crimes by the DNA analysis of slain tissues in the midgut content of larvae, pharmacology, kid, and olds afflicting (5).

Determination of corpse arthropod fauna is one of the basic and inevitable information for estimating of PMI (6). In addition to arthropod fauna, the replacement (succession) of arthropods on corpse can provide important data for PMI estimation in some cases (7, 8). For PMI calculation from less than one week to so many years after death, fundamental information about insect succession plays an important role (9). Successional samples of arthropods on human cadavers are related to the geographic regions of the study areas (2, 10–12). Study on the particular arthropod fauna and stage in various decomposition phases and the relationship between them can be used for estimating the PMI ranges (8).

No recent information on arthropod and insect fauna and their spatio-temporal distribution was available for Tehran, Iran. The aim of the present study was to investigate the fauna of insect species attracted to human cadaver in Tehran district and to acquire knowledge of their temporal and spatial distribution in the region.

## Materials and Methods

Tehran with 18909km^2^ and altitude of 1200 m above sea level, allocates about 2.1% of total area of the country. Tehran has a hot summer, cold winter and brief spring and autumn. This province locates among Mazandaran, Qum, Alborz and Semnan provinces from north, south, west, and east, respectively.

The sampling process was performed during seven months from the beginning of spring to mid autumn 2014.

All procedures were performed in accordance with the terms of the Iran Human (Scientific Procedures) Act Project License and were approved by the Tehran University of Medical Sciences Ethical Review Committee.

This process was done immediately after registering any human corpse in the Kahrizak Autopsy Hall of Tehran Legal Medicine Organization (KAH-TLMO). Among the human cadavers (25–50 per day) referred to the KAH-TLMO, the cases with any kind of arthropods on them were used for arthropod sampling. All of the collected specimens were put individually in labeled vials based on the collection time and sites of the body. The corpse characteristics were recorded including age, sex, location, cause of death, estimated PMI, latitude and longitude, and the arthropod developmental stage (Table 1). To estimate the PMI, medical or scientific evidence other than entomological data such as decomposing phase, body color, decaying of various organs were used to determine PMIs of the corpses. All the vials were transferred to the Medical Entomology Laboratory of School of Public Health, Tehran University of Medical Sciences (SPH-TUMS) in a usual cold chain.

**Table 1.** Details of the human cadavers and the arthropods found on them in Tehran, Iran in 2014

**Case no**	**Gender**	**Age (yr)**	**Location**	**Location**	**Cause of death**	**PMI estimation (d)**	**Development stage**	**Species**
**1**	Male	50	Outdoor	Modares high-way	Unknown	21–28	Larvae	*C. albiceps* *L. sericata*
**2**	Male	68	Indoor	Africa st.	Heart failure	7–10	Larvae	*S. argyrostoma*
**3**	Male	53	Indoor	Dinmohammadi St.	Unknown	14–21	Larvae	*C. albiceps*
							Adult	*M. scalaris*
**4**	Male	60	Indoor	Sattar-khan st.	Unknown	14–21	Larvae	*C. albiceps*
**5**	Male	60 – 70	Outdoor	Saeedi highway	Unknown	30–60	Larvae	*C. albiceps* *L. sericata*
**6**	Male	86	Indoor	Khorassan square	Heart disease	4–7	Larvae	*C. albiceps*
**7**	Male	42	Indoor	Vali-asr st.	Drug abuse	3–4	Larvae	*C. albiceps*
							Egg	*S. argyrostoma*
**8**	Male	65	Indoor	Dastvareh st	Asphyxiation with co	3–5	Larvae	*C. albiceps*
**9**	Female	29	Indoor	Sarbaz st.	Unknown	3–4	Larvae	*S. argyrostoma*
**10**	Male	24	Outdoor	Fasham lacvasan	Hanging on tree	21–28	Pupae	*S. argyrostoma*
**11**	Male	59	Outdoor	Damavand road	Unknown	90–180	Larvae	*D. maculatus*
							Adult	
12*	Male	35	Outdoor	Not defined	Drowned in the lake	Not clear	Larvae	*C. albiceps*

*: The dead body originated from Azerbaijan country.

All the collected specimens were washed with normal detergents and counted. Some of the live immature stages of the arthropod were reared to achieve their adult stage while some of them were killed in boiling water before preserving in 70% EtOH. The specimens were morphologically identified to species level using the known morphological keys (13–18). Main morphological characters used for identifications of the flies were setae on meron, general body color, hairs on greater ampulla, anterior spiracle color, color and hairs on calypters. Whenever it was necessary, the shape and appendages of male genitalia of the specimens were checked to confirm species identification, particularly for Sarcophagidae family. Some of the mature stages of collected arthropods on the corpses were preserved either in 70% EtOH or pinned and deposited in Medical Entomology and Zoology Museum of SPH-TUMS.

## Results

Totally 12 human corpses with arthropod specimens were referred to the Kahrizak Autopsy Hall. Age distribution of the referred corpses was between 23 to 86 years old. Details of the collected human cadavers have been shown in Table 1. Medical or scientific evidence other than entomological data determined PMIs ranged from 3 days to six months for the corpses that could increase arthropod diversities on the cadavers (Table 1).

Various life stages of insects (egg, larvae, and adult) have been observed on the collected cadavers (Table 1). Overall, 4129 arthropod specimens belong to four fly and one beetle species have been collected and identified during the study. The flies comprised two Calliphoridae species of *C. albiceps* (Wiedemann 1819) and *L. sericata* (Meigen 1826), one Sarcophagidae species *S. argyrostoma* (Robineau-Desvoidy 1830), and one Phoridae species *M. scalaris* (Loew 1866). The beetle species was *D. maculatus* DeGeer 1774 (Coleoptera: Dermestidae).

*Chrysomya albiceps* was the dominant species sampled either in outdoor or in indoor adventured human cadavers whereas *L. sericata* and *D. maculatus* was related only to the outdoor places. *Sarcophaga argyrostoma* was related mostly to indoor places. *Megaselia scalaris* was the rarest species due to its merely one specimen in all of the study cases. In outdoor locations, the most representative species were *C. albiceps* (75%,) *L. sericata* (12%), *S. argyrostoma* (3%), and in indoor locations, *C. albiceps* (85%), and *S. argyrostoma* (15%). *Megaselia scalaris* (0.39%) and *D. maculatus* (7%) were collected in smaller numbers in the study area.

## Discussion

In this study, five insect species were observed on human cadavers in Tehran district. Except for *C. albiceps* and *L. sericata*, the other three species including *S. argyrostoma*, *D. maculatus*, and *M. scalaris* are new report for arthropod fauna on human cadavers in Iran. Previous reports have shown presence of various species of blowflies and flesh flies (19–21) on human cadavers. In addition to Calliphoridae and Sarcophagidae, some species of Muscidae and Fanniidae were also involved in human or animal corpse decomposition in Tehran (21).

*Chrysomya albiceps* with 86.09% was the most common species on human cadaver in Tehran districts which is in agreement with the another result (19, 20) in Tehran. The abundance of this species on human corpses reported 58% (19) and 64% (20) which are a little less than that of reported in this study. Usually, *C. albiceps* is one of the most dominant species during hot and dry seasons in other geographical areas (22, 23). This species has been also found dominantly in Schoenly trap equipped with rabbit carcasses in north of Iran (24) and pig carrion in European urban habitats (25). This species has been reported repeatedly in other faunestic studies done with installed meet baited traps in Fars (26) and Tehran (27, 28) Province.

In the present study, *S. argyrostoma* was found as the second foremost species in indoor places. This species is very important in forensic entomology (29) and has been reported repeatedly on animal carcasses (30). This species shows different behaviors on human cadavers in various regions around the world. It has been mentioned mostly as indoor species in Switzerland (31) and Poland (29). Nevertheless, in Germany, this species has been considered as an exclusively outdoor species (32). In this study, this species has been found in one outdoor and three indoor discovered cadavers. At least one of these three indoor cadavers has died certainly at home. This species has also reported in faunestic investigations in the installed meat baited traps in Tehran (28), Fars Province (26) and in Persian Gulf islands (33) and in modified Schoenly trap with rabbit carcasses in north of Iran (24).

We found an individual specimen of *M. scalaris* of Phoridae in indoor location. In indoor places, Phoridae flies can move in rooms with locked doors and windows due to their comparatively small size, and lay their eggs earlier than Calliphoridae (34–36). This makes Phoridae flies a better forensic entomological indicator for estimation of PMI than Calliphoridae larvae in enclosed places and concealed environments (36, 37–39). This species might be the merely criminal entomological evidence accessible if the corpse is blocked or hidden in a habitation that is difficult for other larger insects to gain access (34). Moreover, some species of Phoridae family as well as *M. scalaris* have been mentioned as indicators for buried bodies named coffin fly (34, 40–42). *Megaselia scalaris* has small size and enables to find carrion buried within the ground being found in coffins. They can move through the smallest openings and are able to dig about six feet deep (half a meter in a four-day period) in order to reach buried carrion and lay eggs on carrion to provide nutrition for the emerged larvae (43). However, this species is classified as secondary forensic insect because they favor older rotting cadavers (34). Forensic entomological evidence comprising *M. scalaris* has been used in court as a tool to prove “time of neglect” or lack of care of elderly patients by caretaker (1). Moreover, phorid flies are active in cold season while most blowflies are inactive due to low temperature (42). In cases of myiasis, some phorid species such *M. scalaris* may infest living humans or animals (44, 45).

In the present study, the larvae of beetle *D. maculatus* have been observed on an exposed human cadaver in late decomposition stage. This species also has been reported on human cadavers in Germany (46, 47). Some adults of this species have been trapped in modified Schoenly traps equipped with rabbit carcasses in north of Iran (24). *Dermestes maculatus* is a cosmopolitan species with common name of leather beetle (3). Larvae of this species sometimes act as predator of fly larvae on dead bodies (48). Therefore, observing of this species may not be limited in late stages of decomposition (3). However, this species along with other beetles can be used in forensic investigations especially in late stages of decomposition (49).

Estimation of PMI is urgent and essential for solving the mysteries of death investigations (50). The importance of entomological evidence can be mentioned as well as autopsy (51). Majority of the PMI estimations in the 12 surveyed cadavers in Tehran were more than 3 days. However, lack of accurate PMI estimation was due to lack of entomological investigations in those crime scenes.

## Conclusion

Determination of insect fauna is the first fundamental step for PMI estimation of any specific region. As next step, calculating degree-day requirements and life tables of the species especially *C. albiceps* is highly recommended for establishing forensic entomology. The characters of *C. albiceps* make this species as the best forensic insect candidate for PMI estimation in Tehran and other parts of Iran. Although preliminary studies on temperature requirement of *C. albiceps* has been done in Iran (27) but it warrants to be continued to determine exact life table, behavior, and consistent developmental time of this species alongside with other fly species in various environment situation before establishing PMI in the region.
